# The inhibition activity on tyrosinase, xanthine oxidase, and lipase of *Musa balbisiana* parts grown in Vietnam

**DOI:** 10.1002/fsn3.4364

**Published:** 2024-07-23

**Authors:** Thi Ngoc Nhon Hoang, Le Bao Ngoc Ho, Kim Nhat Nguyet Nguyen, Thi Anh Dao Dong, Thi Hong Anh Le

**Affiliations:** ^1^ Food Science and Technology Faculty Ho Chi Minh City University of Industry and Trade (HUIT) Ho Chi Minh City Vietnam; ^2^ Department of Food Technology, Chemical Engineering Faculty Ho Chi Minh City University of Technology (HCMUT) Ho Chi Minh City Vietnam; ^3^ Vietnam National University Ho Chi Minh City Ho Chi Minh City Vietnam

**Keywords:** lipase, *Musa balbisiana*, tyrosinase, xanthine oxidase

## Abstract

*Musa balbisiana* Colla plant belongs to the *Musaceae* family, which is well‐known for its nutritional and pharmacological properties. This study aimed to evaluate the anti‐xanthine oxidase, anti‐lipase, and anti‐tyrosinase activities of samples of corm, pseudostem, inflorescence, fruit, peel, and seed of *M. balbisiana*. The results show that seed performed the highest capacity in the inhibition of three investigated enzymes, with IC_50_ values of 290.25 μg/mL (anti‐tyrosinase), 141.51 μg/mL (anti‐xanthine oxidase), and 25.66 μg/mL (anti‐lipase). In addition, while fruit showed better performance in anti‐lipase inhibition (IC_50_: 32.72 μg/mL), corm pointed out higher capability on anti‐xanthine oxidase (IC_50_: 160.67 μg/mL) and anti‐tyrosinase (IC_50_: 334.43 μg/mL). The data show the high potential of the application of *M. balbisiana* parts in medical aesthetics, dietary supplements, or medicine. This is the first report to systematically present the biological activities of all parts of *M. balbisiana* grown in Vietnam.

## Graphical abstract

The anti‐xanthanoxidase, anti‐lipase, and anti‐tyrosinase activities of samples from the corm, pseudostem, inflorescence, fruit, peel, and seed of *Musa balbisiana* were determined. The IC50 values of the samples from parts of *M. balbisiana* were 290.25–495.93 μg/mL (anti‐tyrosinase), 141.51–200.78 μg/mL (anti‐xanthine oxidase), and 25.66–47.13 μg/mL (anti‐lipase). The seed sample exhibited the highest inhibition ability of the three investigated enzymes. While fruit showed better performance in anti‐lipase inhibition, corm pointed out a higher capability for anti‐xanthine oxidase and anti‐tyrosinase.

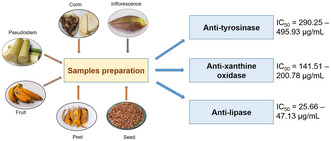

## 1 INTRODUCTION

Natural product treatments have gained a steady increase in interest because of their great potential as a source of lead compounds for certain clinical disorders. Many diseases can be treated with the use of functional foods or edible plants, which are both safe and effective medicinal alternatives for preventing pathophysiological conditions such as diabetes, dyslipidemia, obesity, gout, hypertension, and cancer (Hu et al., 2022). There is a growing demand for natural components from medicine plants to meet the need because customers are worried about the side effects of synthetic products. According to many reports, most of these plants are used as traditional medicines for the treatment of many disorders such as gout and obesity. *Musa balbisiana* is an example. *M. balbisiana* produces rich phytochemical components and secondary metabolites such as flavonoids, polyphenols, tannins, saponins, monoterpenoids, sesquiterpenoids, and quinones in different parts. Thus, parts of the plant are used in ethnomedicinal systems to cure many common diseases (Swargiary et al., 2021). It is necessary to find out or demonstrate the available or new functions of parts of the plant that help to enhance the value of this plant.

In mammalian skin, melanin is known as a major pigment, which is formed by the enzymatic oxidation of tyrosine. It is crucial for safeguarding skin from the harmful effects of ultraviolet (UV) radiation. However, it is also known as a reason for sunburns and mottle. The biosynthesis of melanin is catalyzed by tyrosinase, an enzyme that contains copper. Melanin biosynthesis in mammals, bacteria, plants, and fungi is dependent on this enzyme. The catalytic mechanism is responsible for the rate‐limiting reactions of melanin synthesis: the oxidation of L‐DOPA to o‐dopaquinone and the hydroxylation of tyrosine. The skin's color is influenced by the type and amount of melanin synthesized. The cosmetic and pharmaceutical industries are noticing a great deal of interest in tyrosinase inhibitors because of their prevention of pigmentation disorders and skin aging, or their use in cosmetic products as whitening agents (Masum et al., 2019). Arbutin, glabridin, and kojic acid are well‐known examples, but many worry about synergistic effects. Hence, inhibitors that come from nature have simultaneous requirements to be effective and free from health hazards (Zheng et al., 2008). Thus, the search for more tyrosine inhibitors from natural sources has been ongoing, especially for plants that are rich in bioactive compounds with fewer harmful side effects. Several research studies have identified tyrosinase inhibitors in plants, fungal metabolites, and marine algae.

The abnormally high levels of uric acid in the body are the cause of gout, a metabolic disorder that leads to the formation and deposit of urate crystals in the joints of the lower extremities. Hyperuricemia and gout can be caused by the overproduction or underexcretion of uric acid, which is the end result of purine metabolism in humans. Controlling uric acid levels and reducing or alleviating complications during treatment are important measures for gout patients (Liu et al., 2020). The xanthine oxidase (XO) enzyme plays an important role in the production of uric acid due to the overactivity of xanthine oxidase, which causes high uric acid content. Therefore, xanthine oxidase inhibitors like allopurinol are attractive as potent therapeutic agents due to their ability to block the synthesis of uric acid from purines. Besides, various plant extracts, especially saponins, polyphenols, and flavonoids, are considered for study to inhibit xanthine oxidase (Song et al., 2023). Some studies reported that extracts of some parts of *M. balbisiana*, such as seeds (Irawan et al., 2023), peel (Putri et al., 2022), pulp (Styani et al., 2022), and fruit (Nhon Hoang et al., 2023), showed potential anti‐gout activity. Although synthesis XO inhibitors like allopurinol or benzbromarone appear to have a beneficial effect on experimental animal models and human clinical trials (Britnell et al., 2018), many worry about their side effects in long‐term treatment. Thus, natural XO inhibitors are a growing requirement.

The cause of chronic metabolic disorders is an imbalance between energy intake and expenditure, namely obesity, cancer, endocrine, metabolic, and cardiovascular disorders, which are among the major risk factors (De Pergola & Silvestris, 2013). The pancreatic lipase enzyme is synthesized in the pancreas and is responsible for lipid digestion. Hence, the anti‐lipase factor is one of the most widely studied mechanisms for determining the potential efficacy of natural products as antiobesity agents. Structural changes in the enzyme are responsible for the biochemical activity of the lipase enzyme, which selects and reacts between the lipase binding site and the substrate. Lipase inhibitors have become a significant method for managing and treating obesity, as they control fat metabolism and exhibit low levels of toxicity. Covalent and equimolar lipid–protein complexes are formed when inhibitors react with the nucleophilic serine of the lipase‐active site. The interaction between inhibitors and lipase active sites is able to be reversed. Orlistat is known as a drug acting through pancreatic lipase inhibition. The FDA approves it for the treatment of obesity disorders because orlistat can inactivate the active serine site of lipases to hydrolyze dietary fat by creating a covalent bond with the lipase enzyme (Lunagariya et al., 2014). Synthesis drugs pose potential risks to health because of their side effects. Thus, people tend to use functional foods, edible plants, or plant‐based products as a safe and effective medicine alternative for treating many disorders, including obesity, due to phytochemicals from traditional medicinal plants providing an exciting opportunity for the development of newer therapies. Since the secondary metabolites present in many medical plants have been shown to be lipase inhibitors, among these metabolites, saponins, flavonoids, and alkaloids are present in high concentrations in the plant extract. Their ability to inhibit lipase activity makes them a promising source of lipase inhibitors (Quiroga et al., 2013). Today, a growing concern for medicinal plants has been caused by the increased interest in natural therapeutic approaches for anti‐lipase treatments.

*M. balbisiana* is a popular plant because it is very easy to cultivate and has high tolerance levels in many areas. In Vietnam, the leaves of the plant are often used for wrapping cooked food, for sausages and spring rolls, and in some traditional cakes in the culinary field. The inflorescence is often consumed as a vegetable, while fruits, including the seeds inside, are usually soaked in wine for drinking aimed at medical purposes; the corm, pseudostem, and peel are often discarded. To the best of the authors' knowledge, there were no previous studies on the comparison effects of samples from fractions of all parts of *M. balbisiana* on tyrosine, xanthine oxidase, and lipase inhibition activities. The current study offers an overwhelming overview of the inhibitory ability of lipase, tyrosinase, and xanthinoxidase enzymes in the corm, pseudostem, inflorescence, fruit, peel, and seed of *M. balbisiana* grown in Vietnam. Further pharmacological in vivo studies are required to confirm these findings before practical applications.

## 2 MATERIALS AND METHODS

### 2.1 Materials

**FIGURE 1:**
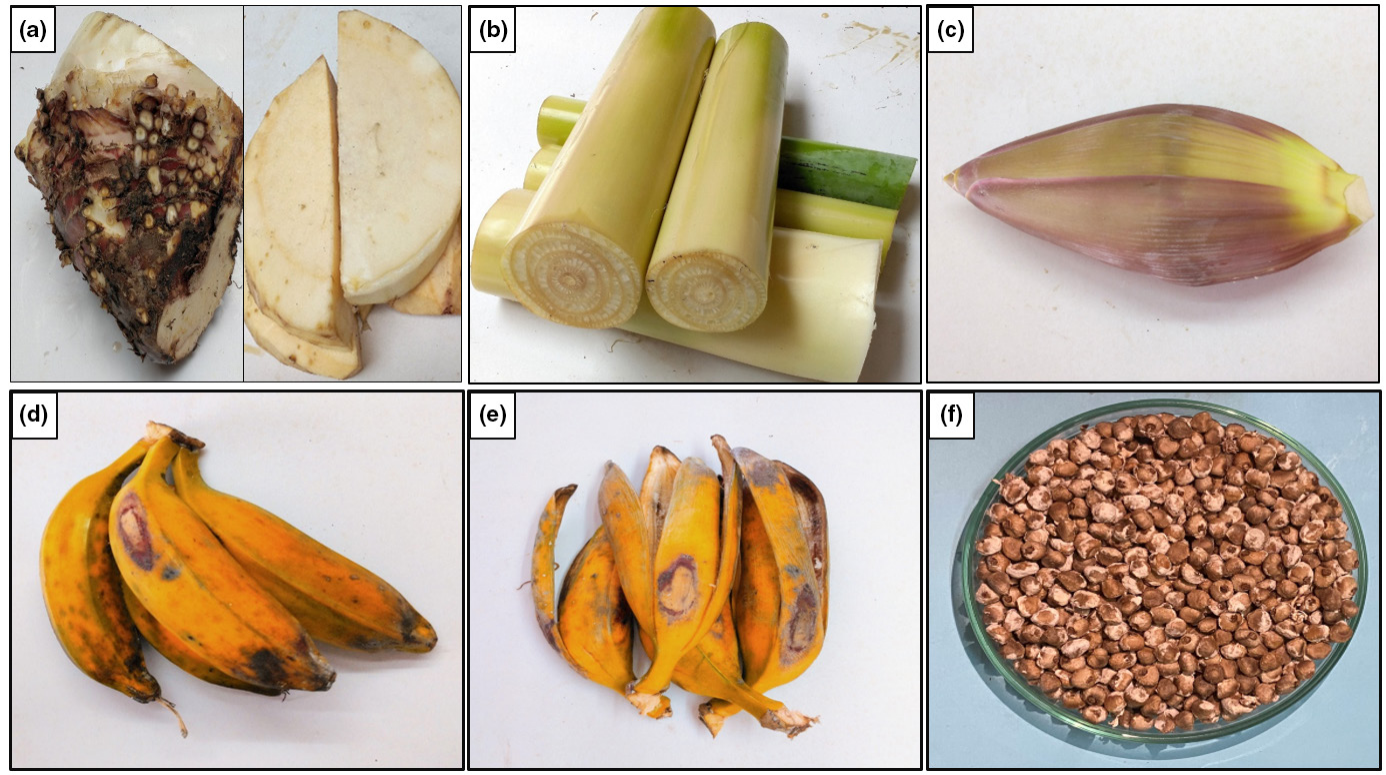
Parts of *Musa balbisiana*: (a) Corm, (b) Pseudostem, (c) Inflorescence, (d) Fruits, (e) Peel, and (f) Seeds.

Fresh *M. balbisiana* parts, namely corm, inflorescence, pseudostem, peel, fruit, and seed, were harvested from An Hoa ward, Tam Nong district, Dong Thap province, Vietnam. Fruits gained about 80–85% maturity were collected from the tree after 115–120 days of blossom. Seeds and peels peaked up from the obtained fruits. The inflorescence was the male flower, which was collected from the tree in full bloom 15 cm away from the bunch of bananas. Corm and pseudostem were collected simultaneously in the same tree after harvesting fruits. To remove the dirt on the surface, these parts were rinsed with tap water and then with distilled water. The pseudostem was kept fresh, while some parts of the corm, inflorescence, peel, and seeds were sliced and dried at 60°C until they were below 10% moisture. They were ground into powder (20–40 mesh size) and stored in zipper bags, away from light, in a refrigerator at a temperature of 4°C for all experiments within 1 month (Figure 1).

Chemicals: L‐DOPA (Sigma), Kojic acid (Sigma), xanthine (Merck), allopurinol (Sigma), lipase enzyme (Sigma), PNPB (p‐nitrophenyl butyrate), Orlistat (Sigma), Tris–HCl (Merck), p‐nitrophenyl butyrate (Merck), lipase (Sigma Aldrich), xanthine oxidase enzyme (Sigma), and mushroom tyrosinase enzyme (Sigma). The chemicals and reagents that are not listed are also of analytical grade.

### 2.2 Methods

#### 2.2.1 Samples preparation

Based on our previous study (Nhon Hoang et al., 2023) and review of the references related to the solvent used in phytochemical extraction (Kibria et al., 2019; Sadat et al., 2017), as well as some preliminary surveys, we chose 70% methanol for the extraction stage. The brief protocol, with some adjustments from references, was summarized. Parts of *M. balbisiana*, including corm, inflorescence, fruit, peel, and seed, were extracted with 70% methanol, the material/solvent ratios 1/20 w/v, and kept in a water bath at 60°C for 120 min. Centrifugation at 5500 rpm for 15 min, followed by filtering through Whatman No. 4 filter paper, was done to ensure homogeneous extracts. Besides, the fresh pseudostem was pressed to have juice. Then, the obtained extracts (corm, inflorescence, fruit, peel, seed) and juice (pseudostem) were continuously partitioned for preliminary purification in a chloroform‐methanol solvent system to eliminate lipids as well as other pigments and select the fractions with high content of total polyphenols (TPC, mg_GAE_/g_dry matter_), total flavonoid content (TFC, mg/g_dry matter_), and saponins (TSC, mg/g_dry matter_) for freeze‐drying to gain samples in powder. The TPC and TSC were evaluated as described in our previous report (Hoang et al., 2023), while the TFC was determined via the UV–Vis method as followed by Fachriyaha et al. (2020). The calibration curves of TPC, TSC, and TFC were *y*_1_ = 0.0111*x* − 0.0319 (TPC, *R* ^2^ = .9989), *y*_2_ = 0.0049*x* − 0.0335 (TSC, *R* ^2^ = .9983), and *y*_3_ = 0.0042*x* − 0.008 (TFC, *R* ^2^ = .9985), respectively. The obtained powder samples of corm, pseudostem, inflorescence, fruit, peel, and seed were stored in PE bags at 4°C for all experiments. The samples were diluted with dimethyl sulfoxide (DMSO) to have different concentrations for the following experiments.

#### 2.2.2 Anti‐tyrosinase activity assay

The tyrosinase inhibitory activities of samples of corm, pseudostem, inflorescence, fruit, peel, and seed against tyrosinase were evaluated by utilizing L‐DOPA as a substrate according to the method of Chen et al. (2010), with minor modifications. The test tubes were filled with 100 L of 100 mM phosphate buffer (pH 6.68) and 20 L of 250 U/mL tyrosine by pipetting. The samples were incubated at 37°C for 20 min after being added at different concentrations (100–500 g/mL). The reaction was started after adding 20 L of 3 mM L‐DOPA to each tube after incubation. After mixing the reaction mixtures with Vortex, the initial absorbance at 490 nm was determined. The IC_50_ is the concentration of samples required for 50% inhibition. Kojic acid was used as the positive control, and the tests were conducted in triplicate. The IC_50_ value indicated up to 50% inhibition of the enzyme. Tyrosinase inhibitory activity was determined by the following equation:

$$\%\text{Inhibition}=\frac{\left(A-B\right)-\left(C-D\right)}{\left(A-B\right)}\times 100\%$$

where *A* is the optical density (OD_490_) of the control; *B* is the OD_490_ of the control with tyrosinase; *C* is the OD_490_ of the test substance; and *D* is the OD_490_ of the test substance without tyrosinase.

#### 2.2.3 Anti‐xanthine oxidase activity assay

Measurement of uric acid generation was used to directly assess the inhibitory properties of xanthine oxidase (XO) with xanthine as the substrate. XO enzyme (0.2 U/mL; 100 μL) was added to a mixture of 0.4 mL potassium phosphate buffer (0.5 mM; pH 7.5) and 100 μL of samples of corm, pseudostem, inflorescence, fruit, peel, and seed of *M. balbisiana* (100–500 μg/mL) and 200 μL of 0.15 mM xanthine. After 30 min of reaction at 37°C, the reaction was halted by adding 200 L of 0.5 M HCl. The measurement of the absorbance at 295 nm was used to determine uric acid production. The blank used was a buffer, and the control was a solution containing xanthine and xanthine oxidase. A positive control is allopurinol (Bondet et al., 1997). The inhibition percentage of xanthine oxidase activity was calculated according to the following formula:

$$\text{Inhibition}\;\left(\%\right)=\frac{A_{\text{control}}-A_{\text{sample}}}{A_{\text{control}}}\times 100\%$$

where *A*_control_ = absorbance of the control and *A*_sample_ = absorbance of the sample.

#### 2.2.4 Anti‐lipase activity assay

The porcine pancreatic lipase inhibitory assay was adapted from AEK Sandeli et al. with minor modifications (Sandeli et al., 2021). The samples stock solution with 100–500 μg/mL was prepared in 10% DMSO. Enzyme stock solution (1 mg/mL): 20 mg lipase enzyme powder was added to 20 mL of 10% DMSO. The preparation of a stock solution of PNPB (p‐nitrophenyl butyrate) involved a dilution of 20.9 mg of PNPB in 2 mL of acetonitrile. Immediately before use, the stock solution of the pancreatic lipase enzyme was prepared. This procedure was carried out on the six studied samples of parts of *M. balbisiana*, namely the corm, pseudostem, inflorescence, fruit, peel, and seed. Next, 0.2 mL extracts with different concentrations were mixed with 0.1 mL of porcine pancreatic lipase (1 mg/mL) and then made up to 1 mL by adding Tri‐HCl solution (pH 7.4) before incubating at 37°C for 30 min. After the incubation period, PNPB solution (0.1 mL) was added to each test tube. A similar procedure was conducted without the extract to obtain a blank solution. The hydrolysis of p‐nitrophenyl butyrate to p‐nitrophenol at 405 nm was determined by measuring it through a UV–visible spectrophotometer. The same procedure was repeated for the other extract and orlistat (a positive control) using the same concentrations as mentioned above. The established tests were performed in triplicates. The lipase enzyme inhibitory activity was measured using the following equation:

$$I\;\left(\%\right)=\frac{A_{\text{blank}}-A_{\text{test}}}{A_{\text{blank}}}$$

where *A*_blank_ = absorbance of the blank and *A*_test_ = absorbance of the sample.

#### 2.2.5 Statistical analysis

The experiments were repeated three times, and the results were analyzed as mean ± SD. Descriptive statistics were used to calculate the mean and standard deviation, and their significant differences (*p* < .05) were evaluated using ANOVA and Tukey's test for multiple comparisons. The variance was assessed by utilizing IBM SPSS Statistics 20 software systems. A one‐way analysis of variance (ANOVA) was used to make statistical comparisons between the normal and treatment groups. Microsoft Excel 2019 software was utilized to draw the charts.

## 3 RESULTS AND DISCUSSION

### 3.1 Anti‐tyrosinase activity

**FIGURE 2:**
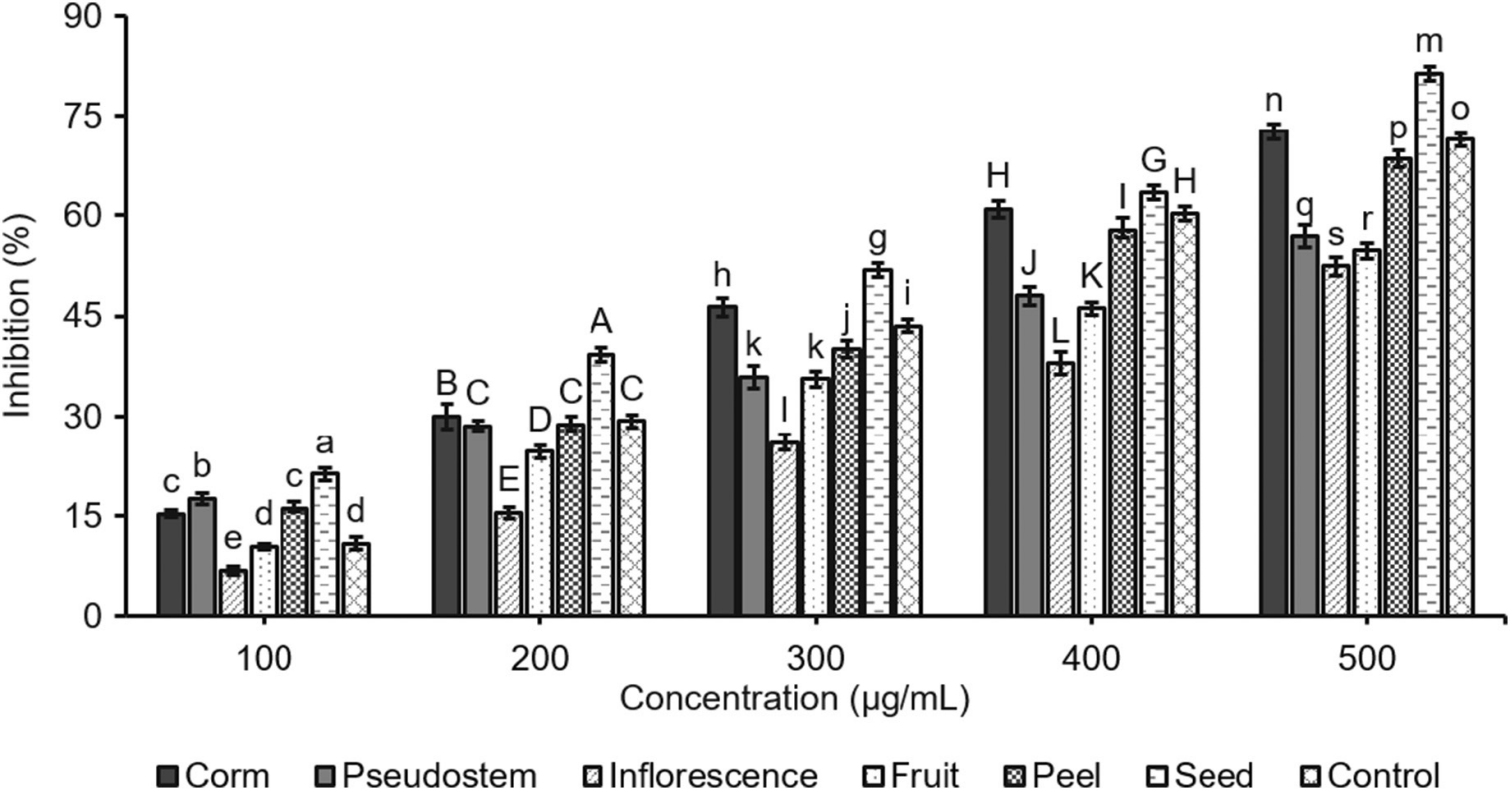
The bar graph of tyrosinase inhibition of the samples from *Musa balbisiana* parts. Different letters, including uppercase and lowercase letters on the bars, represent a statistically significant difference at *p* < .05 according to ANOVA analysis.

Researchers have been interested in finding novel antimelanogenic agents from natural sources to inhibit tyrosinase because tyrosinase plays a vital role in the melanin pathway. These findings have growing importance in medicinal and cosmetic products for treating pigmentation skin disorders. Screening for new inhibitors of melanin biosynthesis involves tyrosinase activity as a significant target because it plays a crucial role in melanogenesis within melanocytes. Melanogenesis is the cause of skin color and serves as a defense against sun‐related injuries. The physical appearance of the skin can be affected by abnormal hyperpigmentation, which can lead to serious esthetic problems like freckles, chloasma, and lentigines (Neagu et al., 2018). Tyrosinase inhibitors could contribute to clinical cures for skin disorders caused by melanin hyperpigmentation. Kojic acid can be utilized as both a skin‐whitening agent and a food additive to stop enzymatic browning. However, many worry about its several side effects. Thus, plant‐based tyrosine inhibitors are attracting interest in cosmetic medicine due to their safety. The effects of the six different parts of *M. balbisiana* on the oxidation reactions of mushroom tyrosinase were assayed by using L‐DOPA as a substrate with kojic acid as a reference compound. L‐DOPA is an inhibitor with the purpose of preventing melanin accumulation in the skin, which is aimed at cosmetics and treatments for pigmentation disorders. The results revealed that all six of the parts inhibited the oxidation of L‐DOPA in a dose‐dependent manner (Figure 2).

**FIGURE 3:**
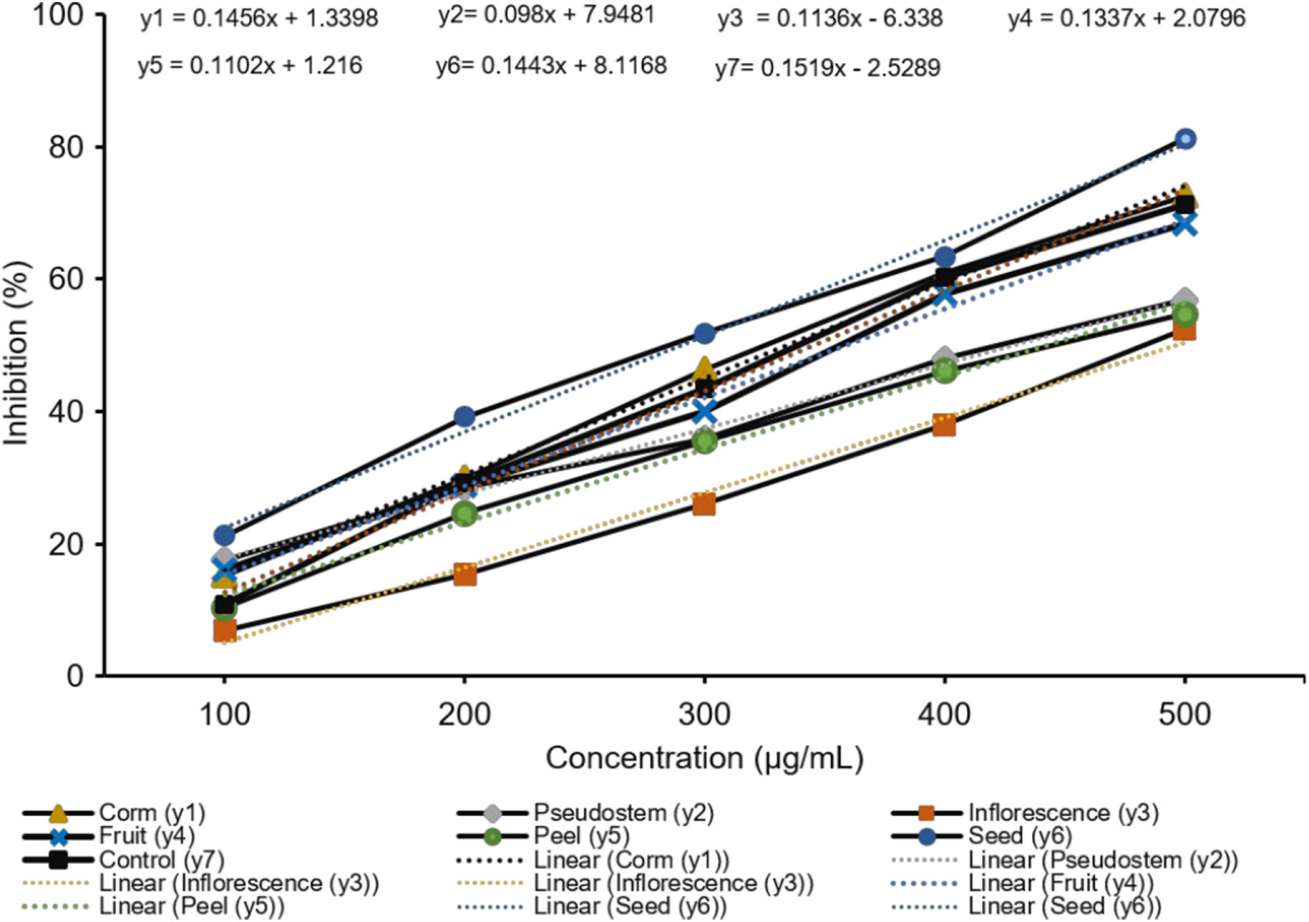
The line graph of tyrosinase inhibition of the samples from *Musa balbisiana* parts.

These results suggest that seed and corm fruit exhibit significant antioxidant and inhibitory activities towards tyrosinase. However, the inhibitory effects of inflorescence, pseudostem, fruit, and peel were less than those of kojic acid. The ability to inhibit the enzyme tyrosinase was dose‐dependent, with an inhibitory effect on tyrosinase activity. IC_50_ is the concentration that shows 50% inhibition. It was calculated from the plot of serial dilutions and the inhibition percentage. The study revealed that the seed sample presented the best tyrosinase activity (IC_50_ 290.25 μg/mL), which is followed in order by samples of corm, fruit, pseudostem, peel, and inflorescence with the IC_50_ values 334.43, 388.49, 429.01, 442.69, and 495.93 μg/mL, respectively. Besides, the value of the control was 345.81 μg/mL (Figure 3). The seed sample concentration of 300 μg/mL showed 51.87% inhibition, the corm sample at 400 μg/mL indicated 60.88% inhibition, and the pseudostem sample at 500 μg/mL performed 56.82%. Therefore, the six samples had noticeable inhibitory effects on the tyrosinase activity. The tyrosinase inhibition activity of parts of *M. balbisiana* was much higher than that of parts of *A. microcarpus* (at 0.2 mg/mL), with the inhibition ranging from 2.25% to 40.25% (Di Petrillo et al., 2016) as well as seed oils derived from the five different varieties of *Torreya grandis* (Cui et al., 2018). The *M. balbisiana* seed and corm samples may be promising herbal preparations containing phytochemicals with significant tyrosinase inhibitors and less toxic side effects. In the cosmetic industry, tyrosinase inhibitors are commonly used to lighten the complexion and treat hyperpigmentary disorders. Additionally, they can be utilized as anti‐browning agents in the food processing industry. In dermatological, biomedical, food, and agricultural science, there is an active field of research on tyrosinase inhibitors from medicinal plants, which have undesirable side effects, as outlined by Mukherjee et al. (2018). Polyphenol and saponin groups of phytomolecules have been proven to be effective in inhibiting tyrosinase. To accurately determine which compounds are responsible for inhibiting this enzyme, it would be necessary to conduct more in‐depth studies that involve the individual components.

### 3.2 Anti‐xanthine oxidase activity

**FIGURE 4:**
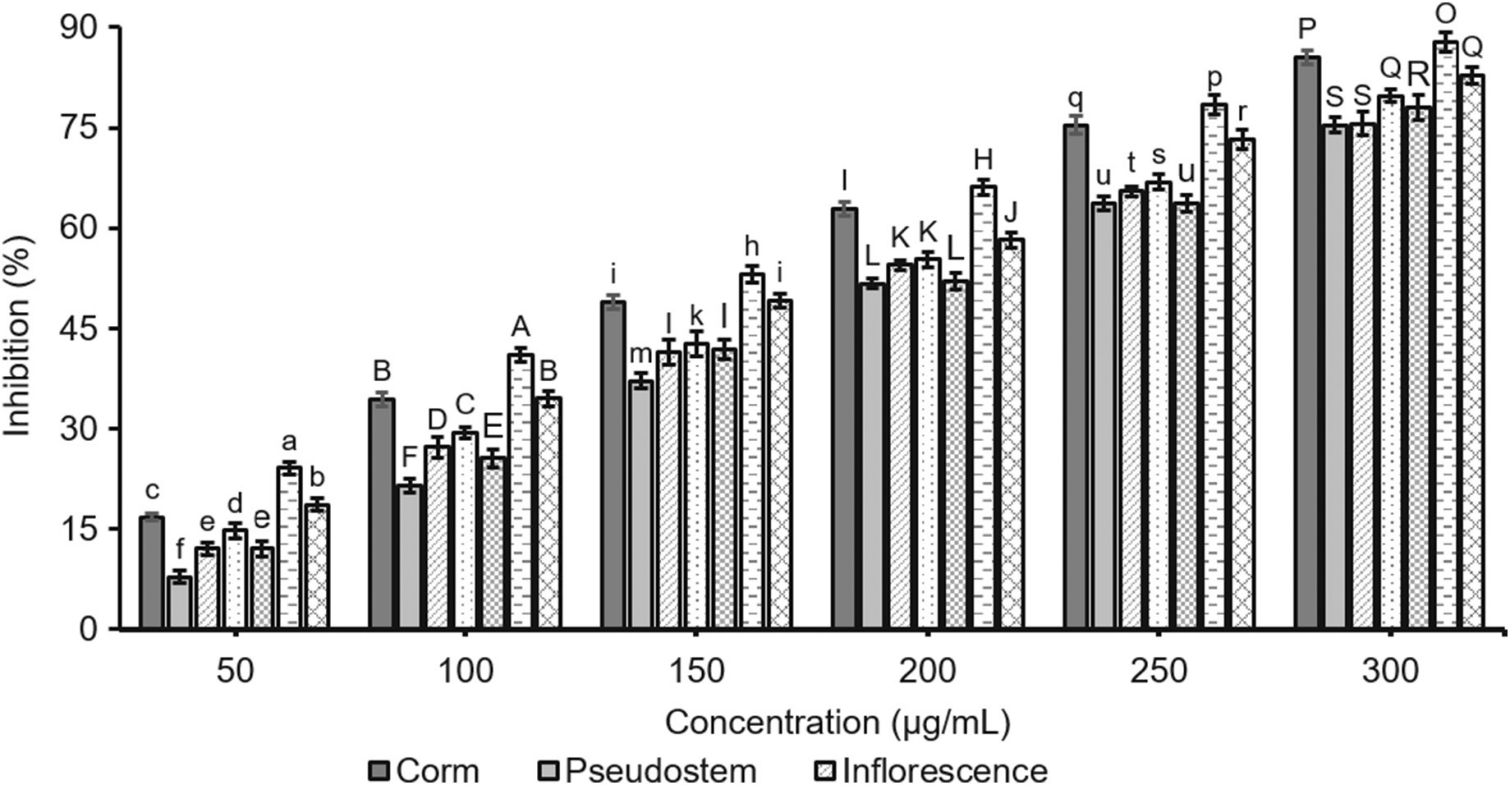
The bar graph of xanthine oxidase inhibition of the samples from *Musa balbisiana* parts. Different letters, including uppercase and lowercase letters on the bars, represent a statistically significant difference at *p* < .05 according to ANOVA analysis.

**FIGURE 5:**
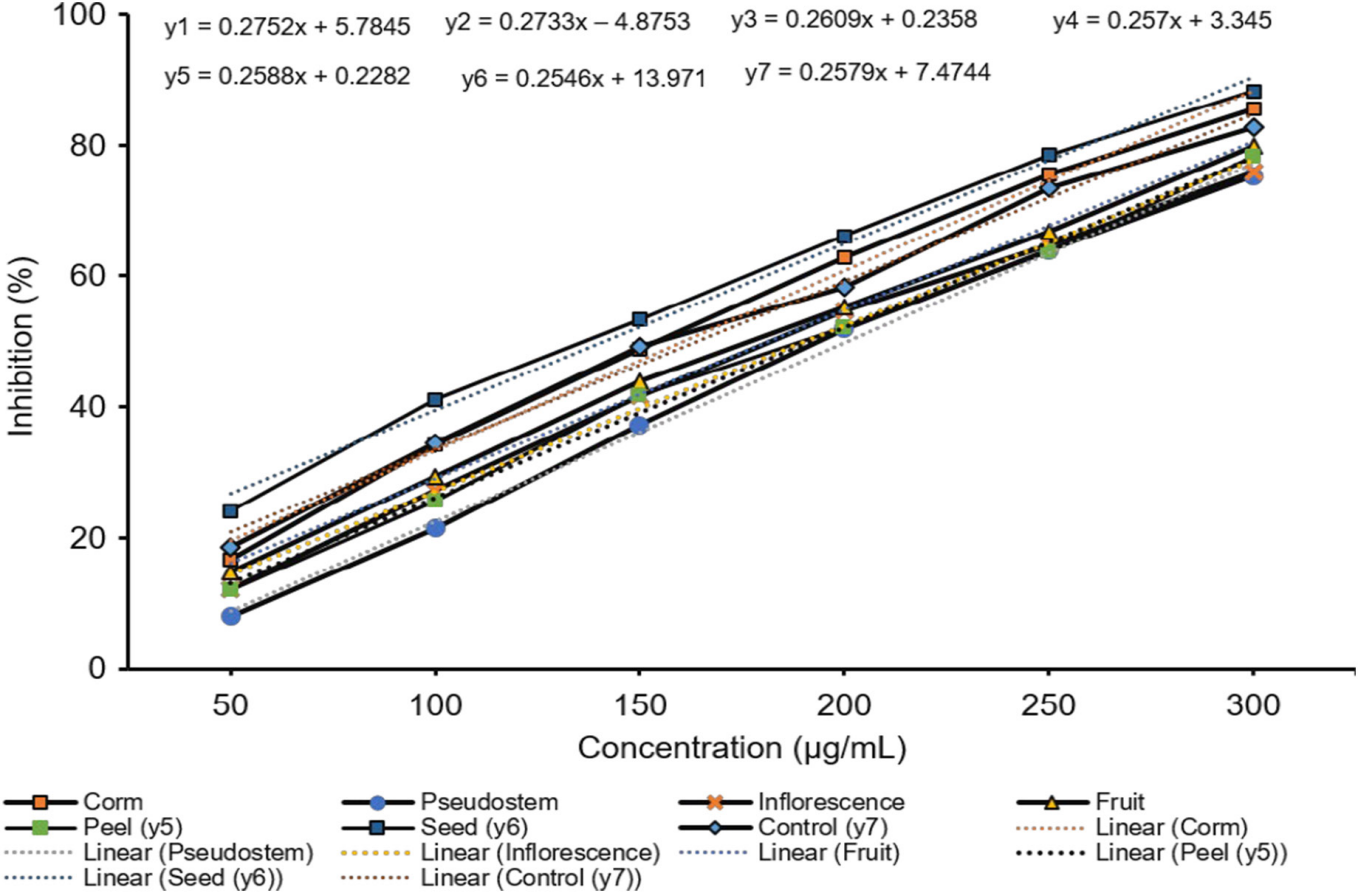
The line graph of xanthine oxidase inhibition of the samples from *Musa balbisiana* parts.

Gout is a disease that affects people worldwide and is caused by the crystallization of uric acid in joints. However, gout is not a common occurrence in patients with hyperuricemia. Several intense gout pains significantly impact patients' quality of life. The consumption of nucleic acid‐rich foods, such as meat, legumes, and some types of seafood, affects the uric acid content. The final step of purine metabolism involves XO, a flavoprotein that oxidizes hypoxanthine to xanthine and generates uric acid, which is essential for gout (Chiang & Chen, 1993). XO inhibitors can block the last stage in uric acid biosynthesis, which lowers the plasma uric acid concentration and is helpful in gout treatment. Besides, XO is also a biological source of oxygen‐derived free radicals, which cause numerous pathological states such as inflammation, ischemia, hepatitis, carcinogenesis, reperfusion, and aging due to oxidative damage to living tissues (Battelli et al., 2014). Thus, studies on natural XO inhibitors with higher therapeutic activity and fewer side effects have been concerning. In this study, the effectiveness of parts of *M. balbisiana* on anti‐xanthine oxidase was determined by an XO inhibition assay and compared with the standard XO enzyme inhibitor, namely allopurinol. The results are indicated in Figures 4 and 5.

The data in Figure 4 indicate that six tested *M. balbisiana* parts exhibited concentration‐dependent inhibitory activity towards the XO. Based on the line graph (Figure 5), the IC_50_ values of the sample of parts allopurinol were 141.51 μg/mL (seed), 160.67 μg/mL (corm), 181.54 μg/mL (fruit), 190.74 μg/mL (inflorescence), 192.32 μg/mL (peel), 200.78 μg/mL (pseudostem), and 164.89 μg/mL (control). In fact, the XO inhibition activity of samples could relate to the total polyphenol, flavonoid, and saponin content of the samples. On the other hand, in comparison to our previous study, the fruit sample showed higher XO inhibition than its extract (Hoang & Dinh, 2023). This might be because the sample in the current study was prepared from fruit extract and then fractioned to eliminate impurities and enhance the bioactive compounds of polyphenols, flavonoids, and saponins. All parts of *M. balbisiana* were rich in saponins and polyphenols, which are known to have many bioactive properties. Saponins and polyphenols are both well‐known antioxidants that can be used as therapeutic agents for diseases caused by free radicals and can effectively inhibit several enzymes, including XO, cyclooxygenase, and lipoxygenase. Saponins and phenolic constituents found in plants have the potential to inhibit XO (Ling & Bochu, 2014). Allopurinol was selected as a positive control because it can reduce uric acid through XO inhibition. Despite its popularity, this drug has serious side effects. Thus, a growing demand for new alternatives with high therapeutic activity and lower side effects has been studied. The XO inhibitory activity of all parts of *M. balbisiana* was higher than the extract and purified fraction from *Pleurotus ostreatus* (from 0.9 to 12.2 mg/mL; Jang et al., 2014), as well as the leaves of *Euphorbia milii*. The methanol extract from the leaves of *E. milii* (*Euphorbiaceae*) with the total phenolic content (0.77 ± 0.02 mg GAE/g of the sample) resulted in an IC_50_ = 0.0864 mM, and it reduced uric acid production by 65.6% (Mutalib et al., 2023).

In short, the results indicate that parts of *M. balbisiana* could inhibit XO in a concentration‐dependent manner, and parts of the seed and corm of this plant were potential sources for XO inhibitors.

### 3.3 Anti‐lipase activity

Recently, people have emphasized the use of functional foods or natural bioactive components from edible plants, particularly traditional medicinal plants. Their toxic effects have been observed and documented since ancient times, as they have been used. This is deemed a safe method to treat obesity and control overweight because it has beneficial health effects and can prevent pathophysiological conditions like obesity, dyslipidemia, diabetes, hypertension, and cancer. The pancreatic lipase enzyme is synthesized and released by the pancreas, responds to lipid digestion, and hydrolyzes about 50–70% of total dietary lipids by splitting triacylglycerols into absorbable monoglycerol and fatty acids. Inhibiting pancreatic lipase is an important strategy in treating obesity (Jaradat, Zaid, Hussein, et al., 2017; Jaradat, Zaid, & Zaghal, 2017). The anti‐lipase activity is studied to determine the effectiveness of natural products as anti‐obesity agents.

**FIGURE 6:**
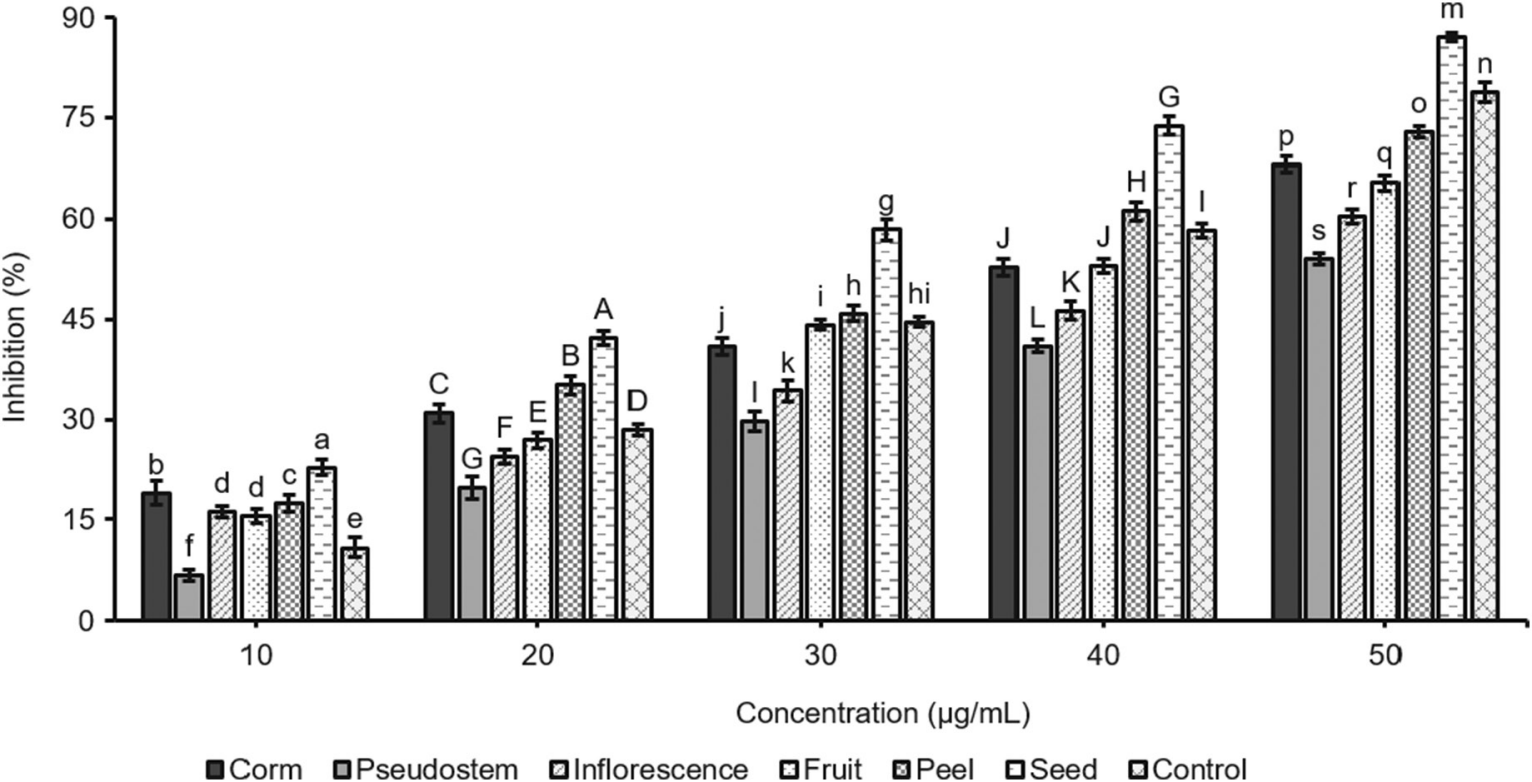
The bar graph of the lipase inhibition activity of the samples from *Musa balbisiana* parts. Different letters, including uppercase and lowercase letters on the bars, represent a statistically significant difference at *p* < .05 according to ANOVA analysis.

**FIGURE 7:**
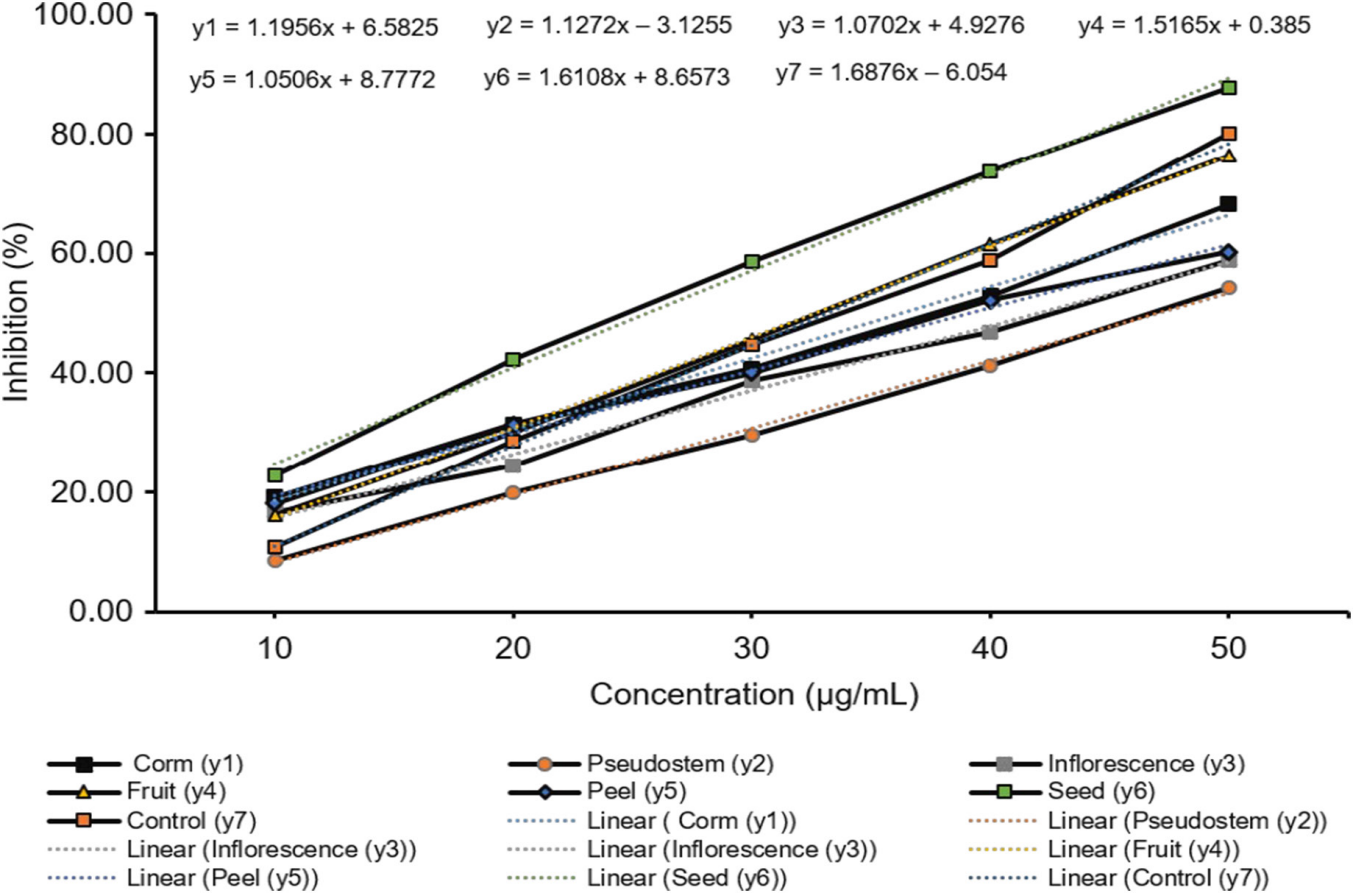
The line graph of lipase inhibition activity of the samples from *Musa balbisiana* parts.

The pancreatic lipase activity of samples of parts of *M. balbisiana* and the positive control of orlistat were evaluated. The inhibitory effects of the reference drug (Orlistat) and samples are dose‐dependent. The IC_50_ values of samples of corm, pseudostem, inflorescence, fruit, peel, and seed were in order 36.31, 47.13, 42.12, 32.72, 39.24, and 25.66 μg/mL. The IC_50_ value of the control was 33.23 μg/mL, respectively (Figures 6 and 7).

The results indicated that the anti‐lipase properties of seed and fruit samples were higher than those of other samples and the control group. The pseudostem, corm, inflorescence, and peel demonstrated lower anti‐lipase activity than orlistat. In detail, the inflorescence sample in the current study showed much stronger anti‐lipase activity in comparison with the flowers of *Musa paradisiaca* collected from Mannargudi, Tamilnadu (Divya et al., 2016). Samples from all parts of *M. balbisiana* also inhibited significantly better than extracts from unripe green fruits (IC_50_ ranged from 1990 to 2347 μg/mL; Sosnowska et al., 2022) and organic and aqueous extracts of *Portulaca oleracea* and *Brassica napus* wild plants from Palestine (IC_50_ between 262.03 and 417.62 μg/mL; Jaradat, Zaid, Hussein, et al., 2017; Jaradat, Zaid, & Zaghal, 2017), sunflower oil and rapeseed oil (IC_50_ were from 53.12 to 188.11 μg/mL; Sosnowska et al., 2016), samples of *Citri Reticulatae Pericarpium* at different harvest times (IC_50_ between 383 and 1370 μg/mL; Zeng et al., 2018) or aqueous and organic extracts of *Arum palaestinum, Crataegu azarolus, Rosmarinus officinalis, Brassica nigra, Taraxacum syriacum* (Jaradat, Zaid, Hussein, et al., 2017; Jaradat, Zaid, & Zaghal, 2017). The samples in this study were prepared from the extracts of parts of *M. balbisiana* and fractions before freeze‐drying. Thus, they determined the anti‐lipase property better than several of the extracts mentioned. Besides, some aqueous and organic extracts from *Vitis vinifera*, *Origanum dayi*, and *Rhus coriaria* showed higher anti‐lipase activity than that of *M. balbisiana* parts (Jaradat, Zaid, Hussein, et al., 2017; Jaradat, Zaid, & Zaghal, 2017). In comparison with parts of *Morus bombycis*, all parts of *M. balbisiana* in this study revealed higher anti‐lipase properties than the parts of the twig, stem, and leaf of *M. bombycis* (IC_50_ values over 100 μg/mL), but lower root extract (IC_50_: 2.07 μg/mL).

In summary, the results of this study indicated that seed and fruit samples of *M. balbisiana* were more effective than a positive control. This promising capability can result from the high content of biocomponents such as saponins and polyphenols in the samples. On the other hand, saponins can perform as emulsifiers to stabilize the interface oil/water emulsions. Thus, saponins can lessen the interaction of lipase with the substrate in terms of aggregating with food fat droplets to form micelles (Karu et al., 2007).

## 4 CONCLUSION

In this study, six selected parts (corm, pseudostem, inflorescence, fruit, peel, and seed) of *M. balbisiana* were investigated for the inhibition of three enzymes: tyrosinase, xanthine oxidase, and lipase. The findings indicate that all parts revealed the potential to inhibit anti‐lipase better than the two other enzymes. The IC_50_ values of the samples from parts of *M. balbisiana* were 290.25–495.93 μg/mL (anti‐tyrosinase), 141.51–200.78 μg/mL (anti‐xanthine oxidase), and 25.66–47.13 μg/mL (anti‐lipase). Among these components, the seeds exhibited the highest level of survey activity, better than the corresponding control. The inhibition of these investigated enzymes might be related to the content of phytochemical compounds in these parts. Current research shows that all parts of *M. balbisiana* possess anti‐tyrosinase, anti‐lipase, and anti‐xanthine oxidase properties, which may be beneficial for the treatment of obesity, skin problems, or gout. However, further research directions, such as in vivo studies, are required to confirm these findings and apply them to pharmaceutical products.

## AUTHOR CONTRIBUTIONS

**Thi Ngoc Nhon Hoang:** Conceptualization (equal); data curation (equal); formal analysis (equal); investigation (equal); methodology (equal); resources (equal); software (equal); validation (equal); writing – original draft (equal); writing – review and editing (equal). **Thi Anh Dao Dong:** Conceptualization (equal); data curation (equal); methodology (equal); supervision (equal); writing – original draft (equal); writing – review and editing (equal). **Thi Hong Anh Le:** Conceptualization (equal); funding acquisition (equal); methodology (equal); supervision (equal); validation (equal); writing – original draft (equal). **Le Bao Ngoc Ho**: Investigation (equal), Data curation (equal). **Kim Nhat Nguyet Nguyen**: Methodology (equal), Investigation (equal).

## CONFLICT OF INTEREST STATEMENT

All authors have read and approved the published version of the manuscript. No conflicts of interest are declared by the authors.

## Data Availability

The data that support the findings of this study are available from the corresponding author upon reasonable request.
